# Association between prepregnancy body mass index and risk of congenital heart defects in offspring: an ambispective observational study in China

**DOI:** 10.1186/s12884-020-03100-w

**Published:** 2020-08-04

**Authors:** Xuelian Yuan, Zhen Liu, Jun Zhu, Ping Yu, Ying Deng, Xinlin Chen, Nana Li, Shengli Li, Shuihua Yang, Jun Li, Hanmin Liu, Xiaohong Li

**Affiliations:** 1 National Office for Maternal and Child Health Surveillance of China, West China Second University Hospital, Sichuan University, Chengdu, Sichuan China; 2 Key Laboratory of Birth Defects and Related Diseases of Women and Children (Sichuan University), Ministry of Education, Chengdu, Sichuan China; 3 Department of Ultrasound, Hubei Maternal and Child Healthcare Hospital, Wuhan, Hubei China; 4 Department of Ultrasound, Shenzhen Maternity and Child Healthcare Hospital, Shenzhen, Guangdong China; 5 Department of Ultrasound, Guangxi Maternal and Child Healthcare Hospital, Nanning, Guangxi China; 6 Department of Ultrasonic Diagnosis, Xijing Hospital, Fourth Military Medical University, Xi’an, Shaanxi China; 7 Department of Pediatrics, West China Second University Hospital, Sichuan University, No. 20 Ren Min Nan Lu, Chengdu City, Sichuan Province 610041 People’s Republic of China; 8 National Center for Birth Defect Monitoring of China, West China Second University Hospital, Sichuan University, No. 17 Ren Min Nan Lu, Chengdu City, Sichuan Province 610041 People’s Republic of China

**Keywords:** Prepregnancy weight, Body mass index, Congenital heart defects, Multilevel logistic regression, Unrestricted cubic spline analysis

## Abstract

**Background:**

Congenital heart defects (CHDs) are the most common birth defect around the world. Maternal prepregnancy obesity has been proposed as a risk factor of CHDs, but the relationship of CHD risk with over- and underweight is controversial, especially because body mass index (BMI) distribution differs between Asia and the West. The study aimed to examine the potential associations of maternal over- and underweight on risk of offspring CHDs.

**Methods:**

An ambispective observational study involving 1206 fetuses with CHDs and 1112 fetuses without defects at seven hospitals in China was conducted. Standardized questionnaires were used to collect information on maternal prepregnancy weight and height, social demographic characteristics, living and occupational environments, and lifestyle behaviors. Univariate, multivariate and multilevel logistic regression as well as unrestricted cubic spline analysis were used to examine potential associations of prepregnancy BMI and offspring CHDs.

**Results:**

Prepregnancy maternal underweight (BMI<18.5) or low average BMI (18.5 ≤ BMI<21.25) was associated with significantly higher risk of CHD in offspring than high average BMI (21.25 ≤ BMI<24.0): multilevel logistic regression indicated adjusted odds ratios of 1.53 (95%CI 1.13, 2.08) for underweight, 1.44 (95%CI 1.10, 1.89) for low average BMI and 1.29 (95%CI 0.84, 1.97) for overweight or obesity (BMI ≥ 24.0). Mothers with prepregnancy BMI < 21.25 were at greater risk of offspring with septal defects, while mothers with low average BMI were at greater risk of offspring with conotruncal defects and septal defects.

**Conclusions:**

Our findings suggest that underweight or low average BMI may be associated with higher risk of CHDs in offspring. Health professionals may wish to advise women planning to be pregnant to maintain or even gain weight to ensure adequate, balanced nutrition and thereby reduce the risk of CHDs in their offspring.

## Background

Congenital heart defects (CHDs) are the most common birth defect, with an estimated prevalence of approximately 9.0 per 1000 live births around the world [1]. CHDs contribute to excess morbidity, premature death, and health-care costs [2, 3]. Genetic factors, infection, phenylketonuria and other factors are known to cause many CHDs [4], but at least 85% of CHDs cannot obviously be attributed to these factors [5]. Therefore, identifying modifiable risk factors of CHDs is important for prevention, and such factors may include drinking, smoking, folic acid intake and prepregnancy body mass index (BMI) [6–8].

Overweight and obesity have been a growing public health concern in developed and developing countries [9]. Prepregnancy obesity in women has been associated with elevated risk of CHDs in offspring [10–13]. More generally, however, there is conflicting evidence about the association of CHD risk in offspring and prepregnancy overweight [10–14] or prepregnancy underweight [13–16].

One reason for this controversy may be related to the different distribution of BMI between women in Western countries and women in Asia, which may reflect differences in genetic, lifestyle, environment, and nutrition diet [17–20]. For example, obesity (BMI ≥ 30) occurs in 30–40% of adult women in developed countries but in only approximately 12.4% of adult women in China [21]. Approximately 20% of women in Southwest Asia and 12.6% of women in China are underweight (BMI<18.5) [21]. A study of 20,321 pregnant women in the Chinese provinces of Sichuan, Yunnan and Guizhou showed prevalence of 18.7% for prepregnancy underweight (BMI<18.5), 67.0% for normal weight (18.5 ≤ BMI<24.0), 12.4% for overweight (24.0 ≤ BMI<28.0), and 1.9% for obesity (BMI ≥ 28.0) [22]. These results suggest the importance of examining potential associations of maternal prepregnancy over- and underweight with offspring CHDs in specific ethnic groups.

To gain insights into these potential associations in Chinese, we undertook a multi-site ambispective observational study involving more than 2300 pregnant women. We examined associations of maternal prepregnancy BMI with risk of single and multiple CHDs in offspring, as well as with risk of specific CHD types.

## Methods

### Study participants

This study recruited pregnant women with a gestational age between 13 and 40 weeks at seven tertiary hospitals with pediatric obstetrics and gynecology wards in Shenzhen, Fuzhou, Wuhan, Zhengzhou, Xian, Chengdu and Nanning between February 2010 and October 2015. All seven hospitals serve as regional centers of genetic counseling, prenatal screening and diagnosis for fetal defects. The study protocol was approved by the Ethics Committee of Sichuan University (2010004), and subjects gave written informed consent. All methods were performed in accordance with the principles of the Declaration of Helsinki.

Among the pregnant women who were willing to undergo prenatal screening and diagnosis via echocardiography, those whose fetus was diagnosed with CHDs and without any abnormalities were initially recruited into case and control group respectively, after obtaining their informed consent. In order to obtain reliable diagnosis results, the diagnosis of each research object was re-confirmed by CHDs cases expert discussion, autopsy, or postnatal examination. Approximately 90% of stillbirths and terminated pregnancies were definitively diagnosed with CHDs based on prenatal ultrasound interpreted by 5–6 specialists and pediatric cardiologists, while the remainder were diagnosed based on autopsy. All the cases of live births were analyzed by ultrasound within the first week after delivery. Controls were confirmed through routine examination and follow-up at 3–6 months after delivery. Pregnant control women were excluded from the study if offspring showed any anomalies.

Women were excluded if (a) their fetus had unclear diagnosis or was diagnosed with other birth defects besides CHDs, (b) they could not recall their prepregnancy weight, or (c) they had diabetes mellitus before or during pregnancy, based on self-report or maternal health records.

### Data collection

Trained staff collected information from pregnant women in face-to-face interviews using a structured questionnaire that asked about maternal demographics and a variety of maternal exposures during the interval from three months before pregnancy until the end of the first trimester. It has been well-described in the published report [23]. These exposures included maternal demographics, home and work environments, lifestyle habits, and pregnancy history. Maternal prepregnancy weight and height were extracted from maternal health records by trained nurses of the hospitals if available; if not, the women were asked to report these data.

### Classification of cases

Cases in our study were coded from Q20 to Q26 on the Atlas of Birth Defects in China [24]. CHDs were classified as septal defect (SPD), conotruncal defect (CTD), left ventricular outflow tract obstruction (LVOTO), right ventricular outflow tract obstruction (RVOTO), anomalous venous return (AVR), or other cardiac structure abnomalities (ELSE) [23, 25]. Subgroup analysis was also performed based on the presence of one or multiple CHDs. In addition, because studies have reported different risks for different septal defect subtypes [12, 26], we examined associations between maternal prepregnancy BMI and risk of ventricular septal defect (VSD) or combined risk of atrial septal or other defects.

### Categories of prepregnancy BMI

Mothers were classified into four groups according to prepregnancy BMI (kg/m^2^): underweight (<18.5), low average BMI (18.5 ≤ BMI ≤ 21.25), high average BMI (21.25<BMI ≤ 23.9), or overweight (≥24.0). Because only 11 subjects had BMI>28.0, which is the typical cut-off for obesity, we assigned them to the overweight group. This categorization is similar to the common classification criterion established by the guidelines for Prevention and Control of Overweight and Obesity in Chinese Adults [27], except that we divided the “normal weight” category into two categories based on the observed median (21.25) in the group of women with average BMI (18.5 ≤ BMI ≤ 23.9). We took this step to better assess trends, given that two-thirds of our subjects showed average BMI. This subdividing of the average BMI group has also been used in studies of the association of prepregnancy BMI with congenital diaphragmatic hernia and CHDs [28, 29].

### Statistical analysis

All analyses were conducted in STATA (Version 15.0; Stata Corp.: College Station, TX, USA). Two-tailed values of P<0.05 and a 95% confidence interval (CI) excluding 1.00 were considered significant, while results associated with two-tailed 0.05 ≤ P<0.10 were considered marginally significant [30]. The factors of residence, maternal age, maternal education, maternal smoking, paternal smoking, maternal drinking, folic acid supplementation and parity were chosed as potential confounders to be analyzed. Differences in frequencies of these factors between cases and controls were assessed using the chi-square test, and factors showing significant differences were adjusted as confounders in subsequent analyses.

Univariate, multivariate and multilevel logistic regression were used to assess associations between CHDs and prepregnancy BMI. Hospital was specified as a random intercept effect in order to isolate influences due to differences in medical facilities. The high average BMI group served as the reference group. To explore a potentially non-linear relationship between offspring CHDs and maternal prepregnancy BMI, restrictive cubic spline analysis was conducted based on the multilevel logistic regression.

## Results

**Fig. 1:**
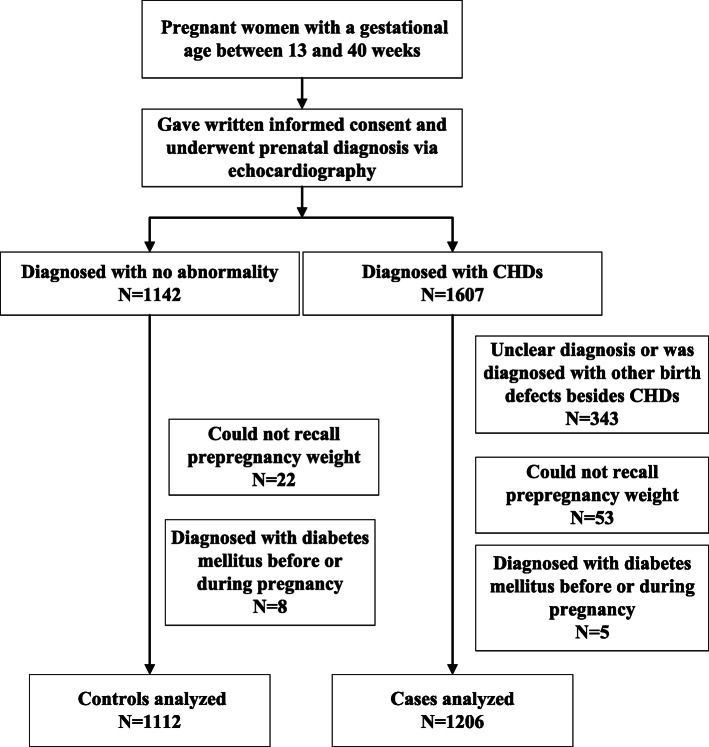
Participant flow chart for the study of prepregnancy BMI of Chinese women and congenital heart defects in their offspring

A total of 2318 women were evaluated for potential inclusion in our study and 431 were excluded (Fig. 1). Cases and controls differed significantly in type of residence, maternal age, education level, maternal smoking, paternal smoking, maternal drinking, parity and folic acid supplementation (Table 1).

**Table 1:** Characteristics of Chinese women with fetuses with or without congenital heart defects (CHDs)

Characteristic	CHD (***n*** = 1206)	No CHD (***n*** = 1112)	P
Residence type ^a^			
urban	731 (62.80)	850 (78.41)	**<0.001**
suburb	183 (15.72)	178 (18.42)	
rural	250 (21.48)	56 (5.17)	
Maternal age ^b^ (yr)			
20–30	894 (74.13)	755 (68.08)	**0.001**
<20 or >30	312 (25.87)	354 (31.92)	
Maternal education			
primary school or below	49 (4.25)	10 (0.92)	**<0.001**
middle school	365 (31.68)	166 (15.24)	
high school	284 (24.65)	268 (24.61)	
university and above	454 (39.41)	645 (59.23)	
Maternal smoking ^c^			
no	1191 (98.76)	1069 (96.13)	**<0.001**
yes	15 (1.24)	43 (3.87)	
Paternal smoking ^c,d^			
no	577 (50.88)	660 (60.05)	**<0.001**
≤ 10 per day	354 (31.22)	284 (25.84)	
≥ 10 per day	203 (17.90)	155 (14.10)	
Maternal drinking ^e^			
no	31 (2.57)	39 (3.51)	**<0.001**
occasional (<1 time/wk)	162 (13.43)	227 (20.41)	
often (≥1 time/wk)	1013 (84.00)	846 (76.08)	
Parity ^f^			
primipara	457 (38.05)	507 (45.68)	**<0.001**
multipara without history of birth defects	728 (60.62)	580 (52.25)	
multipara with history of birth defects	16 (1.33)	23 (2.07)	
Folic acid supplementation ^g^			
no	217 (18.55)	137 (12.40)	**<0.001**
yes	953 (81.45)	968 (87.60)	

Values are n (%) unless otherwise noted. aData missing for 28 controls and 42 cases. bAll the mothers in the study were older than 16 yr. Data missing for 3 controls. c Defined as the person had to smoke throughout the interval from three months before pregnancy until the end of the first trimester. The cut-off of 10 cigarettes per day for fathers corresponded to the median value reported in our sample. dData missing for 13 controls and 72 cases. eBased on the frequency in the interval from three months before pregnancy until the end of the first trimester. Categories were based on previous work [25]. f Data missing for 2 controls and 5 cases. g Maternal supplementation with folic acid or a multivitamin in the interval from three months before pregnancy until the end of the first trimester. Data were missing for 7 controls and 36 cases

Based on the reference group of pregnant women with high average BMI before pregnancy, women who were underweight before pregnancy were more likely to have fetuses with CHDs (multilevel logistic regression OR (mOR) 1.53, 95%CI 1.13, 2.08; Table 2). Similarly, mothers with low average BMI showed higher risk of fetuses with CHDs (mOR 1.44, 95%CI 1.10, 1.89; Table 2).

**Table 2:** Logistic regression to identify interactions between maternal prepregnancy BMI and risk of CHDs in offspring

Subgroup	CHDs n (%)	No CHDs n (%)	cOR(95%CI) ^**c**^	aOR (95%CI) ^**d**^	mOR(95%CI) ^**e**^
All subjects					
BMI < 18.5	333 (27.61)	274 (24.64)	**1.34 (1.06,1.70)**	**1.40 (1.06,1.83)**	**1.53 (1.13,2.08)**
18.5 ≤ BMI < 21.25	540 (44.78)	484 (44.78)	**1.23 (1.00,1.53)**	**1.26 (0.99,1.61)**^**†**^	**1.44 (1.10,1.89)**
21.25 ≤ BMI < 24.00	236 (19.57)	261 (23.47)	Reference	Reference	Reference
BMI ≥ 24.00	97 (8.04)	93 (8.36)	1.15 (0.83,1.61)	1.19 (0.81,1.75)	1.29 (0.84,1.97)
Single CHD ^a^					
BMI < 18.5	191 (26.71)	274 (24.64)	1.25 (0.95,1.65)	**1.39 (1.01,1.92)**	**1.61 (1.12,2.31)**
18.5 ≤ BMI < 21.25	326 (45.59)	484 (44.78)	1.21 (0.95,1.55)	**1.36 (1.02,1.81)**	**1.63 (1.18,2.26)**
21.25 ≤ BMI < 24.00	145 (20.28)	261 (23.47)	Reference	Reference	Reference
BMI ≥ 24.00	53 (7.41)	93 (8.36)	1.03 (0.69,1.52)	1.08 (0.68,1.71)	1.19 (0.71,1.98)
Multiple CHDs ^b^					
BMI < 18.5	142 (28.92)	274 (24.64)	**1.49 (1.09,2.03)**	**1.41 (0.98,2.01)**^**†**^	**1.46 (0.98,2.16)**^**†**^
18.5 ≤ BMI < 21.25	214 (43.58)	484 (44.78)	1.27 (0.95,1.69)	1.11 (0.80,1.55)	1.17 (0.81,1.67)
21.25 ≤ BMI < 24.00	91 (18.53)	261 (23.47)	Reference	Reference	Reference
BMI ≥ 24.00	44 (8.96)	93 (8.36)	1.36 (0.88,2.09)	1.34 (0.81,2.20)	1.34 (0.78,2.30)

^a^All controls and only cases with a single CHD. ^b^All controls and only cases with multiple CHDs. ^c^Crude odds ratio. ^d^Adjusted odds ratio. Data were adjusted for residence, maternal age, maternal education, maternal smoking, paternal smoking, maternal drinking, folic acid supplementation and parity. ^e^Odds ratio from multilevel logistic regression. Odds ratios were adjusted for the factors shown in Table 1, and hospital was set as a random intercept effect. † *P* < 0.10

Of the 1206 babies with CHDs in our study, 715 (59.3%) had a single CHD while 491 (40.7%) had multiple CHDs (Table 2). Subgroup analysis showed that prepregnancy underweight significantly increased risk of a single CHD (mOR 1.61, 95%CI 1.12, 2.31) and multiple CHDs (mOR 1.46, 95%CI 0.98, 2.16; P<0.1), which was a marginally significant effect. Low average BMI was associated with significantly higher risk of a single CHD (mOR 1.63, 95%CI 1.18, 2.26).

We observed a tendency for prepregnancy overweight to increase the risk of CHDs, but the effect did not achieve significance (Table 2). Similar results were obtained when the reference group was set to women with BMI of 19.9 or 22.6, corresponding to the 25th and 75th percentiles of Chinese women in our study with average BMI (18.5 ≤ BMI<24.0; Additional files 1 and 2).

Subgroup analysis by type of CHD indicated that prepregnancy underweight was associated with greater risk of septal defect (SPD) (mOR 1.90, 95% CI 1.11, 3.26), as was low average BMI (mOR 1.63, 95% CI 0.99, 2.67, P<0.10), which was a marginally significant effect. Among the SPD subtypes, risk of prepregnancy underweight was associated with significantly higher risk of VSD (mOR 2.03, 95% CI 1.06, 3.88). Low average BMI was associated with greater risk of conotruncal defect (mOR 1.60, 95% CI 1.01, 2.53) (Table 3).

**Table 3:** Multilevel logistic regression to identify interactions between maternal prepregnancy BMI and subtypes of CHD in offspring ^a^

CHD subtype	Total cases, n	mOR (95%CI) in subgroups based on maternal prepregnancy BMI					
		Subgroup cases, n	Underweight (BMI < 18.5)	Subgroup cases, n	Low average weight (18.5 ≤ BMI < 21.25)	Subgroup cases, n	Overweight (BMI ≥ 24.0)
SPD	238	66	**1.90 (1.11,3.26)**	107	**1.63 (0.99,2.67)**^†^	15	0.96 (0.43,2.13)
VSD	157	48	**2.03 (1.06,3.88)**	70	1.46 (0.81,2.65)	5	0.51 (0.16,1.62)
Other^b^	81	18	1.53 (0.66,3.52)	37	1.58 (0.75,3.33)	10	1.59 (0.56,4.52)
CTD	255	62	1.23 (0.73,2.09)	122	**1.60 (1.01,2.53)**	17	0.83 (0.37,1.88)
LVOTO	82	21	1.52 (0.66,3.54)	37	1.40 (0.64,3.04)	10	1.37 (0.45,4.20)
RVOTO	81	24	1.75 (0.75,4.12)	33	1.33 (0.60,2.93)	7	1.83 (0.60,5.58)
AVR	29	10	2.59 (0.69,9.73)	12	1.87 (0.54,6.52)	3	2.77 (0.53,14.30)
ELSE	30	8	1.63 (0.47,5.71)	15	0.88 (0.26,2.97)	1	0.66 (0.07,6.15)

*SPD* septal defect, *VSD* ventricular septal defect, *CTD* conotruncal defect, *LVOTO* left ventricular outflow tract obstruction, *RVOTO* right ventricular outflow tract obstruction, *AVR* anomalous venous return, *ELSE* other cardiac structure abnormalities. ^a^ Only cases with a single CHD were included. Odds ratios were adjusted for the factors shown in Table 1. Hospital was set as a random intercept effect. ^b^ Cases of the atrial septal defects (*n* = 21) and other septal defects excluding atrial septal defects (*n* = 60) were aggregated because of the small numbers of subjects. ^†^*p* < 0.10

**Fig. 2:**
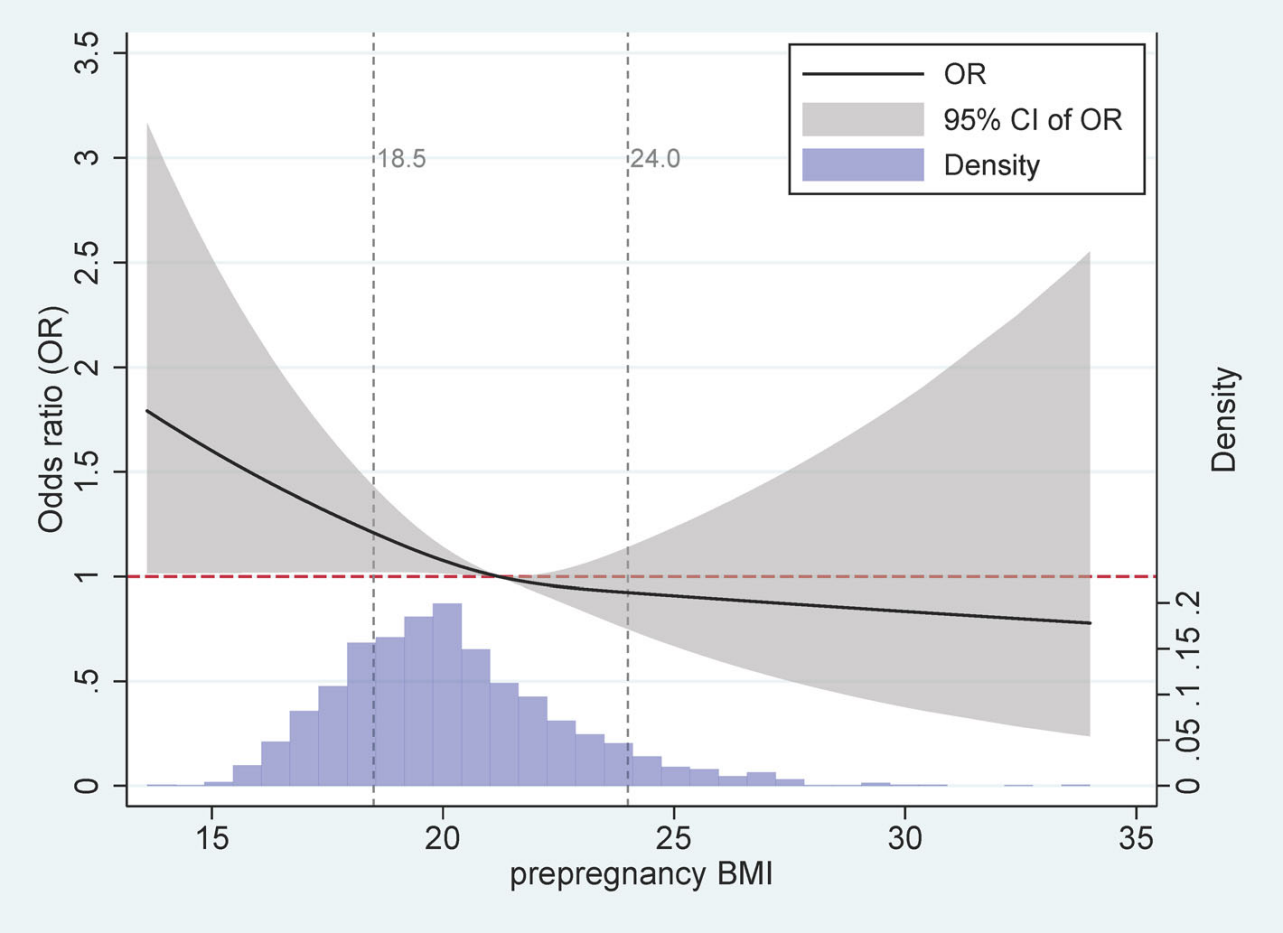
Association between maternal prepregnancy BMI and risk of CHDs in offspring. Odds ratios were adjusted for residence type, maternal age and educational level, maternal smoking, paternal smoking, maternal drinking, folic acid supplementation and parity. Hospital was set as a random intercept effect

Based on the reference group of mothers with prepregnancy BMI of 21.25, which was the median among women with BMI between 18.5 and 24.0, prepregnancy BMI showed an L-shaped relationship with risk of CHDs (Fig. 2). These results are consistent with Table 2. We also found a non-linear relationship of prepregnancy BMI with risk of single or multiple CHDs (Additional files 3 and 4).

## Discussion

We found that prepregnancy BMI showed an L-shaped relationship with risk of CHDs in offspring. Risk of CHDs was significantly higher among mothers with prepregnancy underweight and low average BMI. We failed to observe any significant relationships between prepregnancy overweight or obesity and risk of CHDs in offspring, even after using different cut-off values to define reference groups. This lack of significant association probably reflects the relatively small numbers of mothers in these BMI categories.

Our results are consistent with studies in southeastern and eastern of China, which found that prepregnancy underweight can elevate risk of CHDs in offspring [16, 29]. Similarly to the study in Fujian [29], we divided BMI into groups of BMI < 18.5, 18.5 ≤ BMI < 21.25, 21.25 ≤ BMI < 24.0, and BMI > 24.0, and we found the effect of low average BMI to be associated with higher risk of CHDs in general. The L-shaped relationship that we observed between prepregnancy BMI and risk of general or single CHDs changed to a U-shaped curve when we examined specifically the risk of multiple CHDs. This contrasts with previous work in China showing a U-shaped relationship between prepregnancy BMI and fetal CHDs [16, 29].

For prepregnancy overweight or obesity, some previous studies have found it was associated with a higher risk of offspring CHDs [11, 12]. While, we could not found a statistically significant effect of BMI ≥ 24.0, and it may be due to the relatively small number of overweight women in our study. We did observe a weakly U-shaped correlation between maternal prepregnancy BMI and risk of multiple CHDs in offspring.

How prepregnancy underweight may increase risk of CHDs is unclear. One possibility is malnutrition or nutritional imbalance [31, 32], which can harm embryonic and placental development [33]. Malnutrition or nutritional imbalance can also delay development of the fetal trunk and viscera [31], and cause maternal endocrine abnormalities [29]. In fact, maternal malnutrition or nutritional imbalance can harm fetal organ function even after the organs have fully formed [31]. Some researches have indicated maternal malnutrition or malnutrition imbalance is related with fetal organ dysplasia or malformation, such as: bronchopulmonary dysplasia or neural tube defects [34, 35], while the research of fetal CHDs is few. Future work should examine whether women with different prepregnancy BMI are at risk of malnutrition or micronutrient intake, and whether this in turn affects risk of fetal cardiac anomalies.

Several limitations of the study should be noted. One is that despite the large sample, relatively few subjects were overweight or obese, which may have prevented us from detecting the significant association of such high BMI with CHD risk in offspring that has been reported in studies from the US and Europe [11]. Besides, the very local population in our study may limit the extrapolation of our findings and make direct comparisons to other studies difficult. Therefore we caution against drawing any firm conclusions about these associations from the present study. Another limitation is the design based on retrospective, self-reported data on prepregnancy BMI, which introduces recall bias. We tried to reduce risk of such bias by enrolling women during pregnancy rather than much later. Future work could reduce this bias even more by recording BMI at the first prenatal visit in the first trimester. We were unable to assess whether weight gain during pregnancy influenced risk of CHDs in offspring, since the necessary data were missing for most of our subjects and the gestational weeks for measuring weight were inconsistent, owing that pregnant women do not have regular prenatal examination in the hospitals. Similarly, we did not analyze diet or intake of specific nutrients, which may affect CHD risk. Future work should include these variables. Indeed, our results as a whole should be verified and extended in a prospective cohort study.

## Conclusions

Our analysis of a relatively large sample of women from multiple regions in China suggests that prepregnancy underweight is a risk factor of CHDs in offspring. Our data do not allow firm conclusions about whether prepregnancy overweight or obesity significantly influences CHD risk. Our results should be verified in larger studies, preferably with a prospective cohort. If our findings can be validated, they imply that women planning to get pregnant should maintain or even gain weight in order to maintain an adequate, balanced diet and thereby reduce the risk of CHDs in their offspring.

## Supplementary information

**Additional file 1. **Sensitivity analysis with a reference of BMI 19.9 ≤ BMI<24.0 to examine the interaction between maternal prepregnancy BMI and CHDs in offspring based on logistic regression. ^a^All cases and controls. ^b^All controls and only cases with a single CHD. ^c^All controls and only cases with multiple CHDs. ^d^Crude odds ratio. ^e^Adjusted odds ratio. Data were adjusted for residence, maternal age and educational level, maternal smoking, paternal smoking, maternal drinking, and parity. ^f^Odds ratio from multilevel logistic regression. Data were adjusted for the above potential confounders, and hospital was set as a random intercept effect. ^†^*p* < 0.10.

**Additional file 2.** Sensitivity analysis with a reference of BMI 22.6 ≤ BMI<24.0 to examine the interaction between maternal prepregnancy BMI and CHDs in offspring based on logistic regression. ^a^All cases and controls. ^b^All controls and only cases with a single CHD. ^c^All controls and only cases with multiple CHDs. ^d^Crude odds ratio. ^e^Adjusted odds ratio. Data were adjusted for residence, maternal age and educational level, maternal smoking, paternal smoking, maternal drinking, folic acid supplementation and parity. ^f^Odds ratio from multilevel logistic regression. Data were adjusted for the above potential confounders, and hospital was set as a random intercept effect. ^†^ p < 0.10.

**Additional file 3.** Correlation between maternal prepregnancy BMI and risk of single CHD in offspring. Odds ratios were adjusted for residence type, maternal age and educational level, maternal smoking, paternal smoking, maternal drinking, folic acid supplementation and parity. Hospital was set as a random intercept effect.

**Additional file 4.** Correlation between maternal prepregnancy BMI and risk of multiple CHDs in offspring. Odds ratios were adjusted for residence type, maternal age and educational level, maternal smoking, paternal smoking, maternal drinking, folic acid supplementation and parity. Hospital was set as a random intercept effect.

## Data Availability

Data in our study were collected from seven tertiary hospitals with pediatric obstetrics and gynecology wards. All data are stored electronically in an anonymous format and are currently available only to the main researchers. Data analysis collaborations may be possible on the basis of specific research proposals. Further information can be requested by e-mailing the principal investigator (lixiaohong82@scu.edu.cn).

## Abbreviations

CHDs: Congenital heart defects
BMI: Body mass index
OR: Odds ratios
SPD: Septal defect
VSD: Ventricular septal defect
CTD: Conotruncal defect
LVOTO: Left ventricular outflow tract obstruction
RVOTO: Right ventricular outflow tract obstruction
AVR: Anomalous venous return
