# Supplementary material for: Association between prepregnancy body mass index and risk of congenital heart defects in offspring: an ambispective observational study in China
Source: BMC Pregnancy Childbirth. 2020 Aug 4;20:444. doi: 10.1186/s12884-020-03100-w (PMC7405421; doi:10.1186/s12884-020-03100-w)
Supplement: Supplementary file 2 — Additional file 2. Sensitivity analysis with a reference of BMI 22.6 ≤ BMI<24.0 to examine the interaction between maternal prepregnancy BMI and CHDs in offspring based on logistic regression. aAll cases and controls. bAll controls and only cases with a single CHD. cAll controls and only cases with multiple CHDs. dCrude odds ratio. eAdjusted odds ratio. Data were adjusted for residence, maternal age and educational level, maternal smoking, paternal smoking, maternal drinking, folic acid supplementation and parity. fOdds ratio from multilevel logistic regression. Data were adjusted for the above potential confounders, and hospital was set as a random intercept effect. † p < 0.10. [file 12884_2020_3100_MOESM2_ESM.docx]

| Subgroup | CHDs  n(%) | No CHDs  n(%) | cOR(95%CI)^d^ | aOR (95%CI)^e^ | mOR(95%CI)^f^ |
| --- | --- | --- | --- | --- | --- |
| All subjects^a^ |  |  |  |  |  |
| BMI＜18.5 | 333 (27.61) | 274 (24.64) | 1.40 (1.00,1.96) | **1.44 (0.98,2.13)**^†^ | **1.60 (1.03,2.47)** |
| 18.5≤BMI＜22.6 | 696 (57.71) | 653 (58.72) | 1.23 (0.89,1.68) | 1.24 (0.86,1.78) | 1.38 (0.93,2.08) |
| 22.6≤BMI＜24.0 | 80 (6.63) | 92 (8.27) | Reference | Reference | Reference |
| BMI≥24.0 | 97 (8.04) | 93 (8.36) | 1.20 (0.79,1.81) | 1.23 (0.77,1.98) | 1.36 (0.80,2.29) |
| Single CHDs^b^ |  |  |  |  |  |
| BMI＜18.5 | 191 (26.71) | 274 (24.64) | 1.43 (0.95,2.13) | **1.61 (1.01,2.57)** | **1.83 (1.08,3.12)** |
| 18.5≤BMI＜22.6 | 426 (59.58) | 653 (58.72) | 1.33 (0.91,1.94) | **1.49 (0.96,2.30)**^†^ | **1.69 (1.03,2.77)** |
| 22.6≤BMI＜24.0 | 45 (6.29) | 92 (6.29) | Reference | Reference | Reference |
| BMI≥24.0 | 53 (7.41) | 93 (8.36) | 1.17 (0.71,1.90) | 1.25 (0.71,2.21) | 1.37 (0.72,2.60) |
| Multiple CHDs^c^ |  |  |  |  |  |
| BMI＜18.5 | 142 (28.92) | 274 (24.64) | 1.36 (0.88,2.11) | 1.16 (0.70,1.92) | 1.26 (0.73,2.19) |
| 18.5≤BMI＜22.6 | 270 (54.99) | 653 (58.72) | 1.09 (0.72,1.64) | 0.87 (0.54,1.41) | 0.96 (0.57,1.60) |
| 22.6≤BMI＜24.0 | 35 (7.13) | 92 (8.27) | Reference | Reference | Reference |
| BMI≥24.0 | 44 (8.96) | 93 (8.36) | 1.24 (0.73,2.11) | 1.11 (0.61,2.05) | 1.16 (0.60,2.26) |
