# Supplementary figures and images for: Association between prepregnancy body mass index and risk of congenital heart defects in offspring: an ambispective observational study in China
Source: BMC Pregnancy Childbirth. 2020 Aug 4;20:444. doi: 10.1186/s12884-020-03100-w (PMC7405421; doi:10.1186/s12884-020-03100-w)

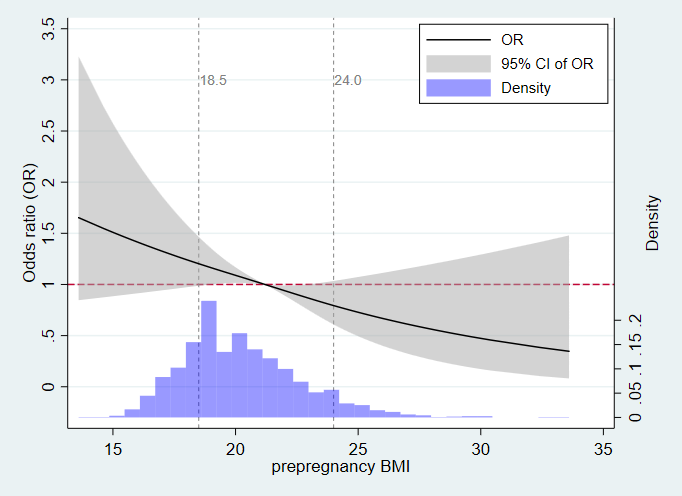

Supplement: Supplementary file 3 — Additional file 3. Correlation between maternal prepregnancy BMI and risk of single CHD in offspring. Odds ratios were adjusted for residence type, maternal age and educational level, maternal smoking, paternal smoking, maternal drinking, folic acid supplementation and parity. Hospital was set as a random intercept effect. [file 12884_2020_3100_MOESM3_ESM.docx]

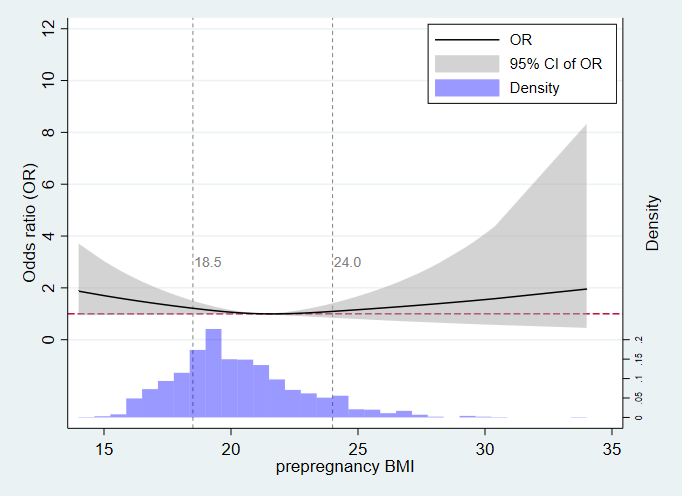

Supplement: Supplementary file 4 — Additional file 4. Correlation between maternal prepregnancy BMI and risk of multiple CHDs in offspring. Odds ratios were adjusted for residence type, maternal age and educational level, maternal smoking, paternal smoking, maternal drinking, folic acid supplementation and parity. Hospital was set as a random intercept effect. [file 12884_2020_3100_MOESM4_ESM.docx]
